# Association of N-Terminal Pro-Brain Natriuretic Peptide with Cognitive Function and Depression in Elderly People with Type 2 Diabetes

**DOI:** 10.1371/journal.pone.0044569

**Published:** 2012-09-04

**Authors:** Insa Feinkohl, Naveed Sattar, Paul Welsh, Rebecca M. Reynolds, Ian J. Deary, Mark W. J. Strachan, Jackie F. Price, on behalf of the Edinburgh Type 2 Diabetes Study (ET2DS) Investigators

**Affiliations:** German Diabetes Center, Leibniz Center for Diabetes Research at Heinrich Heine University Duesseldorf, Germany

## Abstract

**Background:**

Type 2 diabetes mellitus is associated with risk of congestive heart failure (CHF), cognitive dysfunction and depression. CHF itself is linked both to poor cognition and depression. The ventricular N-terminal pro-brain natriuretic peptide (NT-proBNP) is a marker of CHF, suggesting potential as a marker for cognitive impairment and/or depression. This was tested in the Edinburgh Type 2 Diabetes Study (ET2DS).

**Methodology and Principal Findings:**

Cross-sectional analysis of 1066 men and women aged 60–75 with type 2 diabetes. Results from seven neuropsychological tests were combined in a standardised general cognitive ability factor, ‘g’. A vocabulary-based test estimated pre-morbid cognitive ability. The Hospital Anxiety and Depression Scale (HADS) assessed possible depression. After adjustment for age and sex, raised plasma NT-proBNP was weakly associated with lower ‘g’ and higher depression scores (ß −0.09, 95% CI −0.13 to −0.03, p = 0.004 and ß 0.08, 95% CI 0.04 to 0.12, p<0.001, respectively). Comparing extreme quintiles of NT-proBNP, subjects in the highest quintile were more likely to have reduced cognitive ability (within the lowest tertile of ‘g’) and ‘possible’ depression (HADS depression ≥8) (OR 1.80; 95% CI: 1.20, 2.70; p = 0.005 and OR 2.18; 95% CI: 1.28, 3.71; p = 0.004, respectively). Associations persisted when pre-morbid ability was adjusted for, but as expected were no longer statistically significant following the adjustment for diabetes-related and vascular co-variates (β −0.02, 95% CI −0.07 to 0.03, p>0.05 for ‘g’; β 0.03, 95% CI −0.02 to 0.07, p>0.05 for depression scores).

**Conclusion:**

Raised plasma NT-proBNP was weakly but statistically significantly associated with poorer cognitive function and depression. The prospective phases of the ET2DS will help determine whether or not NT-proBNP can be considered a risk marker for subsequent cognitive impairment and incident depression and whether it provides additional information over and above traditional risk factors for these conditions.

## Introduction

People with type 2 diabetes are at around 1.5 to 2.5-fold increased risk of developing dementia [1], [2], a disorder involving progressive cognitive, behavioural and motor deficits. Type 2 diabetes is also associated with an increased risk of age-associated cognitive decline, short of frank dementia [3], and with depression [4], [5], a condition closely related to cognitive dysfunction. The underlying mechanisms responsible for these links are unclear.

Co-morbidity with congestive heart failure (CHF) is also prevalent in diabetes [6], [7]; there appears to be a poorly defined bidirectional relationship between cardiac function and glucose metabolism. Cardiac stress initiates secretion of natriuretic peptides from the ventricles [8], including N-terminal pro-brain natriuretic peptide (NT-proBNP) the inactive metabolite of the proBNP hormone. Higher circulating NT-proBNP concentrations in populations with diabetes compared with healthy adults have been reported [9], [10].

There is some evidence in predominantly non-diabetic populations to suggest that BNP (with which circulating NT-proBNP is highly correlated [11]) is associated with poor cognitive ability. Raised natriuretic peptide concentrations have been related to low performance on measures screening for poor global cognitive functioning potentially indicative of dementia [12]–[14]. Raised natriuretic peptide concentrations are also found in cognitively impaired individuals [15], [16] and may correlate with severity of dementia [14], [17]. In the only prospective investigation to date, proBNP predicted dementia at 5-year follow-up [18].

Evidence also indicates raised NT-proBNP in individuals with major depressive disorder (MDD) and correlations between NT-proBNP and number of depressive symptoms in healthy populations [19] and in those with cardiovascular disease [20]–[22]. Further, left ventricular dysfunction has been associated with poor cognition [23]–[25] and depression [25]. However, overall evidence is inconclusive, with several studies failing to find a statistically significant association of NT-proBNP with depression or quality of life [26]–[29], with cognitive function [30], [31], or dementia [15].

Given these findings, NT-proBNP could function as a biomarker of accelerated cognitive aging and depression in people with type 2 diabetes. High prevalence of cognitive and mood disorders in type 2 diabetes offers an interesting and novel opportunity to study the relationship between natriuretic peptides, acting as a proxy for cardiac stress, and cognitive function and depression. The present study aims to assess the association of NT-proBNP with level of cognitive ability, with level of ability relative to estimated peak pre-morbid ability, and with depressive symptoms, in a large, representative cohort of elderly patients with the full spectrum of severity of type 2 diabetes (the Edinburgh Type 2 Diabetes Study, ET2DS).

## Methods

### Study Population

Data from the cross-sectional, baseline phase of the prospective ET2DS were analysed. Details of the recruitment and examination for the ET2DS have been reported previously [32]. In brief, in 2006/7, 1066 community-dwelling men and women with type 2 diabetes mellitus, aged 60 to 75 years and living in the Lothian area of Scotland, UK, were selected at random from a comprehensive register of patients with diabetes attending primary or secondary care in this region. Subjects underwent detailed physical and cognitive examinations in a dedicated research clinic by specially trained nurses using standard operating procedures.

### Ethics Statement

Assessments complied with the ethical rules of the Declaration of Helsinki. Ethical approval for the study was obtained from the Lothian Medical Research Ethics Committee, and all participants gave written informed consent.

### Physical Examination

Participants were assessed by self-completion questionnaire and physical examination for a range of demographic and clinical characteristics. Diabetes-related measurements included plasma HbA1c, fasting glucose, duration of diabetes and mode of treatment. Vascular measurements included systolic and diastolic brachial blood pressure, body mass index (BMI), waist-hip ratio (WHR), % body fat, smoking status, alcohol intake, total serum cholesterol and ankle brachial index (ABI). ABI is the ratio of systolic blood pressure in the ankle to the systolic blood pressure in the arm, and has been used as a measure both of peripheral arterial disease and more generalised subclinical atherosclerosis [33], [34]. Pre-specified criteria were used to define prior myocardial infarction (MI), angina or stroke using a combination of self-report of a doctor diagnosis, WHO chest pain questionnaire, 12-lead ECG and linkage to hospital discharge records, as has been described in detail previously [35].

### Cognitive and Mood Assessment

Seven neuropsychological tests measured various dimensions of cognitive ability. The Borkowski Verbal Fluency Test (BVFT) examined executive function. Immediate and delayed recall was assessed in the Logical Memory subtest (LM) of the Wechsler Memory Scale 3rd Edition (WMS-III). The Faces subtest of the WMS-III was a measure of non-verbal memory. Mental flexibility and visual attention were examined by the Trail-Making Test B (TMT-B). Several subtests of the Wechsler Adult Intelligence Scale 3rd Edition (WAIS-III) were administered: Digit Symbol Coding (DSC; speed of information processing), Letter Number Sequencing (LNS; working memory) and the Matrix Reasoning test (MR; non-verbal reasoning). Vocabulary (‘crystallised’ intelligence) was measured using the combined junior and senior Mill Hill Vocabulary scale (MHVS) synonyms. As the results of vocabulary tests show very little mean decline with ageing, they can be used to estimate peak, prior cognitive ability [36]. When late-life cognitive ability scores are adjusted for vocabulary-based test scores, the residuals correlate highly with actual measures of cognitive change over a lifetime [37]. A score of below 24 out of 30 on the Mini-Mental-State Examination (MMSE; [38]) indicated presence of possible dementia. Presence of depression and anxiety was assessed on the Hospital Anxiety and Depression Scale (HADS; [39]). Responses were made on four-point scales, with maximum scores of 21 on each of two subscales, the HADS-A and HADS-D. In addition to the use of the HADS-D as a continuous outcome, ‘possible cases’ of clinical depression (HADS-D ≥8) were identified. Both these applications of the HADS are widely employed in the literature [40], and the cut-point of 8 has been shown to have high sensitivity and specificity [41]. A majority of participants completed the entire cognitive test battery and mood assessment (n = 1048 to 1065 for individual tests), and provided data on all physical measures (n = 1028 to 1066). Incompleteness was mainly due to physical difficulty rather than test refusal.

### Measurement of NT-proBNP

Fasting blood samples were taken at the research clinic, and plasma was frozen for storage. Plasma NT-proBNP concentrations were determined using the Elecsys 2010 electrochemiluminescence method (Roche Diagnostics, Burgess Hill, UK) calibrated using the manufacturer's reagents. Manufacturer's controls were used with limits of acceptability defined by the manufacturer. Low control CV was 6.7% and high control CV was 4.9%.

### Statistical Methods

Distribution of values was skewed for NT-proBNP, HADS-D, and TMT-B. These variables were transformed to their natural logarithm values.

Individuals who perform well on one cognitive test tend to perform well on another, so that different cognitive tests load on a single common factor, ‘*g*’ [42]. Principal component analysis revealed that all seven cognitive tests (LM, Faces, MR, DSC, TMT-B, LNS, BVFT) could indeed be captured by the single standardised factor, ‘*g*’.

Initial two-tailed Pearson correlations tested associations of NT-proBNP with other clinical measures, cognitive outcome and HADS depression scores. Mean NT-proBNP levels were compared between groups with suspected dementia (MMSE<24), and between those with suspected clinical depression (HADS-D score ≥8) and those without.

In exploratory univariate analyses, mean ‘*g*’ and HADS depression scores were compared between the highest and the lower four quintile NT-proBNP groups. The likelihood of low cognitive functioning (scoring in the lowest versus intermediate or highest tertiles of ‘*g*’) and of suspected clinical depression were calculated for the highest quintile NT-proBNP as an example of a group with raised NT-proBNP levels.

Scores on the different cognitive tests were also treated as individual continuous outcome variables. NT-proBNP was modelled on each of these, ‘*g*’, and HADS depression, adjusting for potential confounders in staged and cumulative linear regression analyses. Confounders were selected on the basis of results from exploratory univariate analyses and reports in the literature of confounding or mediating roles in the relationship of NT-proBNP with cognition or depression. Results from the regression analyses were presented as β regression coefficients to show the direction and strength of the associations, signifying the change in cognitive and depression scores for each unit increase in NT-proBNP.

For cognitive outcome variables, only age and sex were controlled for in a first model. Estimated pre-morbid cognitive ability (MHVS) was entered in a second stage, before the addition of diabetes-related and cardiovascular risk factor variables (smoking status, blood pressure, BMI, HbA1c, cholesterol and mode of treatment). MI, angina, stroke and ankle brachial index (ABI) were then entered, but had not previously been included in order to avoid over-adjustment. HADS depression scores were added in a final model in order to determine the potential independence of NT-proBNP associations with cognition and depression. The first two modelling steps were also followed for the HADS depression outcome. In a third step, HbA1c and mode of treatment were controlled for. Cardiovascular disease variables were only added into the model in a final step, again in order to avoid over-adjustment given the role of NT-proBNP as a marker of cardiovascular disease. Analyses were performed using SPSS for Windows, version 14.0 (IBM Corporation, New York).

## Results

### Cohort characteristics and classical risk factors

Characteristics of the ET2DS participants are shown in Table 1. The study population (n = 1066) has been shown previously to be largely representative of all people invited to participate in the study (n = 5454) and therefore of the target population of older men and women with type 2 diabetes living in the general population [35]. Mean age was 67.9 years. Mean plasma NT-proBNP levels were similar in men and women (geometric means 79 pg/ml ±3 vs 84 pg/ml ±3; p = 0.41 and correlated positively with age (r = 0.25; p<0.001). Controlling for age and sex, NT-proBNP was significantly associated with the following variables: duration of diabetes (r = 0.14; p<0.001), diastolic blood pressure (r = −0.09; p = 0.008), ABI (r = −0.14; p<0.001), BMI (r = 0.11; p<0.001) and cholesterol (r = −0.10; p = 0.005), but not with HbA1c, plasma glucose or systolic blood pressure (p>0.05). Mean levels differed between individuals with MI, stroke or angina and those without (p<0.001 respectively), but not between smokers and non-smokers (p>0.05).

**Table 1:** Baseline characteristics of the ET2DS population.

Variable	Mean ± SD, median (quartile range) or n (%)
**Age (years)**	67.9±4.2
**Male sex (%)**	547 (51.3)
**Duration of diabetes (years)**	6 (3–11)
**Treatment of diabetes with insulin +/− tablets**	186 (17)
**Treatment with tablets alone**	679 (64)
**Treatment with dietary change alone**	200 (19)
**HbA1c (%)**	7.4±1.1
**Fasting plasma glucose (mmol/L)**	7.6±2.1
**Systolic BP (mmHg)**	133±16
**Diastolic BP (mmHg)**	69±9
**Myocardial infarction**	150 (14)
**Angina**	298 (28)
**Stroke**	62 (6)
**ABI**	0.98±0.21
**BMI (kg/m^2^)**	31.4±5.7
**Total cholesterol (mmol/L)**	4.3±0.9
**Smoking (current) (%)**	153 (14.4)
**NT-proBNP (pg/ml)**	75 (37–169)
**Hospital Anxiety and Depression Questionnaire (HADS): Depression score**	3 (1–6)
**HADS depression score ≥8**	78 (7.3)
**Mini-Mental State Examination <24/30 (%)**	30 (2.9)
**Trail-Making Test B**	104 (81–138)
**Matrix Reasoning**	12.8±5.3
**Digit Symbol Coding**	49.2±14.8
**Verbal Fluency Test**	36.9±12.8
**Letter Number Sequencing**	9.7±2.8
**Faces**	65.8±7.9
**Logical Memory**	25.2±8.2
***g***	0.00±1.00
**Mill Hill Vocabulary Scale**	30.9±5.2

Mean ± SD is given for normally distributed variables.

Median (quartile range) is given for non-normally distributed variables.

### NT-proBNP associations with cognitive performance

After controlling for age and sex, NT-proBNP was weakly but statistically significantly correlated with decreased performance on MHVS, Logical Memory, TMT-B, DSC, MR, the general ability factor ‘*g*’ and HADS depression. NT-proBNP levels accounted for <1% of variance in cognitive test scores and ‘*g*’ (r^2^ = 0.005 to 0.008), and for 1.7% of depression scores. (r^2^ = 0.017; Table 2). NT-proBNP was not significantly different in the small group of participants with possible dementia (MMSE<24, n = 30) compared with higher scoring individuals (geometric mean 106 pg/ml ±3 vs 81 pg/ml ±3, p = 0.24). However, the group with possible clinical depression (HADS depression score ≥8, n = 78) had lower mean MMSE scores (geometric means 27.80±1.08 vs 28.28±1.08, p = 0.052, Cohen's *d* = 0.24) and higher NT-proBNP levels (geometric mean 107 pg/ml ±3 vs 80 pg/ml ±3, p = 0.03, Cohen's *d* = 0.28) compared to the non-depressed group.

**Table 2:** Age and sex-adjusted two- tailed partial correlations of NT-proBNP with cognitive and depression scores.

	Partial correlation coefficient	p-value
**Mill-Hill Vocabulary Scale**	−0.07	0.042
**Borkowski Verbal Fluency**	<0.01	0.963
**Logical Memory**	−0.08	0.015
**Faces**	−0.05	0.139
**ln(Trail-Making-Test-B)**	0.08	0.012
**Digit Symbol Coding**	−0.09	0.006
**Letter Number Sequencing**	−0.05	0.146
**Matrix Reasoning**	−0.08	0.010
***g***	−0.09	0.007
**ln(HADS depression)**	0.13	<0.001

To further explore whether plasma NT-proBNP could be a marker of reduced cognitive function irrespective of other possible markers, we examined differences in cognitive ability across quintiles of NT-proBNP (geometric cutpoints were 29.70, 59.15, 100.48 and 217.02 pg/ml). An analysis of covariance with adjustment for age and sex suggested an overall trend across quintiles (adjusted mean ‘g’ = 0.02, 0.14, 0.13, −0.09 and −0.18 respectively; p = 0.003). Subjects in the highest quintile of NT-proBNP (n = 199) had lower ‘g’ than those in the remaining four quintiles (n = 807; adjusted means −0.18 versus 0.05; 95% CI for difference −0.39, −0.08; p = 0.003). In unadjusted analyses comparing the highest quintile of NT-proBNP to all other quintiles, the odds ratios for low cognitive ability (‘g’ in the lowest tertile) was 2.14 (95% CI: 1.44, 3.18; p<0.001). After adjustment for age and sex, the association was marginally attenuated to 1.80 (95% CI: 1.20, 2.70; p = 0.005).

For ‘g’ and each of the constituent cognitive tests, multiple regression analyses were carried out (Table 3). The initial model controlled for age and sex. Associations with NT-proBNP reached statistical significance for a number of cognitive tests (Logical Memory, TMT-B, DSC, MR), and ‘g’. In a second step, pre-morbid cognitive ability (MHVS) was added into the model. The associations with TMT-B, DSC, MR, and ‘g’ remained statistically significant, and for Logical Memory approached significance. When a range of potential confounding and/or mediating variables, including conventional cardiovascular risk factors together with HbA1c and treatment type were added in a third model, only the association with Logical Memory remained statistically significant and also survived the further adjustment for previous cardiovascular disease and HADS depression in a final step.

**Table 3:** Multivariate associations between NT-proBNP and cognitive test scores.

		MHVS	g	BVFT	LM	FACES	ln(TMT-B)	DSC	LNS	MR
**Model 1a**	**β**	−0.26	−0.09	0.02	−0.49	−0.31	0.03	−0.96	−0.10	−0.35
	**95% CI**	−0.54, 0.01	−0.13, –0.03**	−0.65, 0.69	−0.91, −0.06*	−0.71, 0.10	0.01, 0.05*	−1.71, −0.20**	−0.25, 0.04	−0.62, −0.08**
**Model 2b**	**β**		−0.05	0.28	−0.36	−0.20	0.02	−0.69	−0.04	−0.25
	**95% CI**		−0.09, 0.00*	−0.33, −0.89	−0.75, 0.04	−0.59, 0.19	0.00, 0.04*	−1.38, 0.00*	−0.18, 0.90	−0.50, −0.01*
**Model 3c**	**β**		−0.04	0.38	−0.48	−0.15	0.02	−0.26	−0.04	−0.23
	**95% CI**		−0.08, 0.01	−0.26, 1.01	−0.89, −0.07*	−0.56, 0.26	0.00, 0.04	−0.98, 0.46	−0.17, 0.10	−0.48, 0.02
**Model 4d**	**β**		−0.02	0.18	−0.57	−0.01	0.02	0.19	−0.01	−0.15
	**95% CI**		−0.07, 0.03	−0.52, 0.87	−1.02, −0.11*	−0.46, 0.44	−0.01, 0.04	−0.60, 0.98	−0.16, 0.15	−0.43, 0.13
**Model 5e**	**β**		−0.02	0.16	−0.56	−0.03	0.01	0.26	0.01	−0.14
	**95% CI**		−0.06, 0.03	−0.56, 0.88	−1.03, −0.08*	−0.50, 0.44	−0.01, 0.04	−0.54, 1.07	−0.15, 0.16	−0.42, 0.15

The β scores are regression coefficients.

a adjusted for age and sex.

b adjusted for +MHVS.

c adjusted for + smoking status, blood pressure, BMI, HbA1c, cholesterol, mode of treatment.

d adjusted for + MI, angina, stroke, ABI.

e adjusted for + ln(HADS depression).

* significant at 0.05 level.

** significant at 0.01 level.

### NT-proBNP associations with depression

Adjusting for age and sex, HADS depression scores gradually increased across NT-proBNP quintiles (geometric mean scores were 2.77, 2.94, 3.19, 3.25 and 3.56 respectively; p = 0.007). The highest NT-proBNP quintile had higher mean depression scores compared with the lower four quintiles (95% CI 0.05, 0.28; p = 0.006). The age- and sex-adjusted odds ratio (OR) for possible clinical depression, comparing the highest versus the lower four quintiles, was 2.18 (95% CI 1.28, 3.71; p = 0.004).

Multivariable modelling was repeated for HADS depression. The age and sex adjusted association reached significance and survived additional adjustment for pre-morbid cognitive ability, as well as diabetes-associated variables. The association became non-significant following the addition of previous cardiovascular disease into the model (Table 4).

**Table 4:** Multivariate associations between NT-proBNP and depression scores.

		ln(HADS depression)
**Model 1** a	**β**	0.08
	**95% CI**	0.04, 0.12**
**Model 2** b	**β**	0.08
	**95% CI**	0.04, 0.12**
**Model 3** c	**β**	0.07
	**95% CI**	0.03, 0.11*
**Model 4** d	**β**	0.03
	**95% CI**	−0.02, 0.07

The β scores are regression coefficients.

a adjusted for age and sex.

b adjusted for +MHVS.

c adjusted for + HbA1c, mode of treatment.

d adjusted for + MI, angina, stroke, ABI.

* significant at 0.05 level.

** significant at 0.01 level.

## Discussion

This epidemiological study investigated the contribution of NT-proBNP levels to performance on a large battery of cognitive tests and symptoms of depression in older people with type 2 diabetes using one of the largest community-based study populations on this topic. Cognitive ability, measured by the standardised factor ‘*g*’, was lower, and depression higher, in patients with raised plasma NT-proBNP. Participants with peptide levels in the highest quintile of NT-proBNP distribution were almost two times more likely to have reduced cognitive ability (lowest tertile of ‘*g*’) and were more than two times more likely to have possible clinical depression following the adjustment for age and sex, compared with the remaining population, but associations of NT-proBNP with ‘*g*’ and depression scores no longer reached statistical significance when diabetes-related and vascular co-variates were additionally controlled for.

Although not the main focus of the present investigation, raised NT-proBNP was also found to be associated with poorer pre-morbid cognitive ability. This finding could cast doubt over a causal relationship between cardiac stress (as measured by natriuretic peptides) and late-life cognitive ability in people with diabetes, since ‘reverse causation’ and confounding are possible explanations [43]. Low early life cognitive ability leads to lower late-life ability, and exposes individuals to poor lifestyle choices, health problems with associated raised biomarker levels, and earlier death [44]. However, the reported weak associations between NT-proBNP and current cognitive ability remained significant when pre-morbid ability was controlled for.

The association between reduced late-life cognitive ability and plasma NT-proBNP is consistent with previous studies on predominantly non-diabetic populations [12]–[14]. However, previous studies have predominantly used simple screening instruments, such as the MMSE, to measure cognitive function. Our use of a general ability factor derived from a number of different cognitive tests is unique in the literature on NT-proBNP and cognition. Although the age- and sex-adjusted odds of reduced cognitive ability for the high NT-proBNP group were comparable to those reported by Daniels et al. [12], the multivariate associations between NT-proBNP and cognitive scores were relatively weak compared with some previous investigations [13], [14]. Peptide levels were also similar in subjects with and without suspected dementia determined using the MMSE (MMSE<24) compared with others. This finding is consistent with previous studies [15], [30], [31], but may reflect flaws in the use of the MMSE, designed to screen for dementia, as a measure of milder forms of cognitive impairment [45].

Associations between NT-proBNP and deficits in specific cognitive domains were also explored. When age and sex were controlled for, peptide levels were unrelated to performance on BVFT, Faces or LNS, but were associated with poor performance on Logical Memory, TMT-B, DSC and MR. Furthermore, these relatively weak associations (except that for Logical Memory, for which a trend was observed) remained statistically significant following adjustment for estimated pre-morbid ability. The finding of inverse associations between peptide levels and performance on the TMT-B test of visual attention and the Matrix Reasoning test of non-verbal reasoning is consistent with investigations in non-diabetic populations [12], [14]. NT-proBNP was unrelated to verbal fluency (BVFT) both in the present study and in Gunstad et al. [14]. Associations in Daniels et al. [12] also only reached statistical significance when analyses were largely unadjusted. NT-proBNP has not previously been investigated in relation to nonverbal memory (Faces), speed of information processing (DSC) or working memory (LNS), of which we found an association only with speed of processing. It must be noted that since a large number of analyses were carried out, it is possible that the statistically significant associations arose through type I error. Further studies are required to confirm or refute these findings.

For any disease, the identification of biomarkers indicating increased risk of complications and co-morbidities is vital to allow identification of high risk individuals. Despite high prevalence of vascular disease and associated high average NT-proBNP levels in people with type 2 diabetes [9], [10], as well as high prevalences of cognitive and mood disorders, to our knowledge, this is the first investigation to focus on the relationship of NT-proBNP with cognitive function and mood in type 2 diabetes.

The association of natriuretic peptides with late cognitive ability may well reflect underlying cardiac stress and associated vascular disease, rather than any direct causal relationship. This is supported by the attenuation of the relationship between cognitive ability and NT-proBNP following the adjustment for a range of cardiovascular risk factors and cardiovascular disease, which rendered it statistically non-significant. A weak association between NT-proBNP and the Logical Memory test (which measures verbal declarative memory through recall of a short story and is particularly sensitive to progression of type 2 diabetes [46]) was unique, in that it was not attenuated by the adjustment for cardiovascular or diabetes-associated variables, or HADS depression scores. Despite the possibility that the result represents a type I error, it is an intriguing finding which merits further investigation.

Raised NT-proBNP was also associated with high depression scores, supporting previous findings from non-diabetic populations [19]–[22]. The relative weakness of the associations is consistent with one previous report [22], although associations overall tend to be larger [19]–[21]. Despite predicting high risk of future vascular disease and depression, low pre-morbid ability did not confound the association. Pathways linking depression and NT-proBNP levels have been suggested previously [19]. We found evidence against this, as the association became non-significant when previous cardiovascular disease was controlled for. Depression is associated with a harmful lifestyle and subsequent endothelial damage, and also accelerates the progression of cardiac illness through effects on the hypothalamic-pituitary adrenal (HPA) axis, increasing the risk of heart failure [47]. Conversely, heart failure and other chronic illness initiate symptoms of depression, including hypersomnia, fatigue and loss of energy [48], constructs included in the HADS questionnaire.

Overall, the clinical relevance of natriuretic peptides as biomarkers for poor cognitive function and depression is unclear. Despite a relatively weak multivariate association, individuals in the highest NT-proBNP quintile were substantially more likely to have reduced cognitive ability and depression compared with the remaining population. NT-proBNP may function as a biomarker of subclinical cerebrovascular disease particularly in individuals who experience cardiac stress. This extends its well-established role as a predictor of cardiovascular and cerebrovascular events [49]. Mirroring a greater ability of natriuretic peptides compared with other biomarkers, such as c-reactive protein (CRP), to predict these outcomes [50], the association of NT-proBNP with cognitive function in the present study was also stronger than that of CRP with cognitive function in a previous analysis [35].

A strength of the current study was the ability to adjust for a wide variety of variables, both those which are commonly controlled for (e.g., age), and those which may be confounding or mediating factors underlying the relationship between NT-proBNP and cognitive ability (e.g., vascular risk factors; cardiovascular disease). Due to the large size of the study, results are more robust than those of previous investigations carried out on smaller scales [15]–[17] and the analysis had high power to detect even weak associations, although admittedly, the clinical relevance of such associations is debatable. The latter was reflected in the conservative interpretation of statistically significant results. The cross-sectional nature of the study and the resulting unclear direction of the reported associations is a weakness of the study, although this was partly offset by our adjustment for pre-morbid cognitive ability. The MHVS as a measure of pre-morbid ability is advantageous over the often used level of education, which is subject to self-report bias.

In conclusion, we found some evidence for NT-proBNP as a biomarker of low cognitive ability and depression in elderly people with type 2 diabetes, although the associations were relatively weak and were largely explained by previous cardiovascular disease. The value of NT-proBNP measurement over and above the assessment of traditional markers of cognitive dysfunction and depression remains unclear, and further studies on its relationship with these disorders are needed. Analysis of the second wave of data of the prospective ET2DS will provide an ideal opportunity to investigate the associations of natriuretic peptide levels with risk of subsequent cognitive decline and the development of depression.

## References

[1] Biessels GJ, Staekenborg S, Brunner E, Brayne C, Scheltens P (2006) Risk of dementia in diabetes mellitus: a systematic review. Lancet Neurol: 64–74. 10.1016/S1474-4422(05)70284-2 16361024

[2] Cukierman T, Gerstein HC, Williamson KD (2005) Cognitive decline and dementia in diabetes- systematic overview of prospective observational studies. Diabetologia 48: 2460–2469. 16283246 10.1007/s00125-005-0023-4

[3] Bourdel-Marchasson I, Lapre E, Laksir H, Puget E (2010) Insulin resistance, diabetes and cognitive function: consequences for preventative strategies. Diabetes Metab 26: 173–181. 10.1016/j.diabet.2010.03.001 20472485

[4] Ali S, Stone MA, Peters JL, Davies MJ, Khunti K (2006) The prevalence of co-morbid depression in adults with type 2 diabetes: a systematic review and meta-analysis. Diabet Med 23: 1165–1173. 17054590 10.1111/j.1464-5491.2006.01943.x

[5] Anderson RJ, Freeland KE, Clouse RE, Lustman P (2001) The prevalence of comorbid depression in adults with diabetes. Diabetes Care 24: 1069–1078. 11375373 10.2337/diacare.24.6.1069

[6] He J, Ogden LG, Bazzano LA, Vupputuri S, Loria C, et al (2001) Risk factors for congestive heart failure in US men and women. Arch Intern Med 161: 996–1002. 11295963 10.1001/archinte.161.7.996

[7] Nichols GA, Gullion CM, Koro CE, Ephross SA, Brown JB (2004) The incidence of congestive heart failure in type 2 diabetes. Diabetes Care 27: 1879–1884. 15277411 10.2337/diacare.27.8.1879

[8] Doust JA, Glasziou PP, Pietrzak E, Dobson AJ (2004) A systematic review of the diagnostic accuracy of natriuretic peptides for heart failure. Arch Intern Med 164: 1978–1984. 15477431 10.1001/archinte.164.18.1978

[9] Andersen NH, Poulsen SH, Knudsen ST, Heickendorff L, Mogensen CE (2005) NT-proBNP in normoalbuminuric patients with type 2 diabetes mellitus. Diabet Med 22: 188–195. 15660737 10.1111/j.1464-5491.2004.01396.x

[10] Magnusson M, Melander O, Israelsson B, Grubb A, Groop L, et al (2004) Elevated plasma levels of NT-proBNP in patients with type 2 diabetes without overt cardiovascular disease. Diabetes Care 27: 1929–1935. 15277419 10.2337/diacare.27.8.1929

[11] Castleberry C, Lo S, Tillman K, Stendahl G, Ahn J, et al (2010) NT-proBNP versus BNP and their correlation with cardiac function in pediatric transplant of heart failure patients. J Am Coll Cardiol 55: A43.E410.

[12] Daniels LB, Chen W-C, Kritz-Silverstein D, Laughlin GA, Maisel AS, et al (2009) Elevated NT-proBNP levels are associated with poor cognitive function in apparently healthy older adults: results from the Rancho Bernado Study. Circulation 120: S542.

[13] Mauro F, Rosso GL, Peano M, Agostini M, Aspromonte N, et al (2007) Correlation between cognitive impairment and prognostic parameters in patients with congestive heart failure. Arch Med Res 38: 234–239. 17227734 10.1016/j.arcmed.2006.10.004

[14] Gunstad J, Poppas A, Smeal S, Paul RH, Tate DF, et al (2006) Relation of brain natriuretic peptide levels to cognitive dysfunction in adults >55 years of age with cardiovascular disease. Am J Cardiol 98: 538–540. 16893713 10.1016/j.amjcard.2006.02.062 PMC2748274

[15] Kondziella D, Goethlin M, Fu M, Zetterberg H, Wallin A (2009) B-type natriuretic peptide plasma levels are elevated in subcortical vascular dementia. Neuroreport 20: 825–827. 19424098 10.1097/WNR.0b013e328326f82f

[16] Suwa M, Ito T (2009) Correlation between cognitive impairment and left ventricular diastolic dysfunction in patients with cardiovascular diseases. Int J Cardiol 136: 351–354. 18684536 10.1016/j.ijcard.2008.04.099

[17] Naito J, Naka Y, Watanabe H (2009) Clinical impression of brain natriuretic peptide levels in demented patients without cardiovascular disease. Geriatr Gerontol Int 9: 242–245. 19702933 10.1111/j.1447-0594.2009.00526.x

[18] Kerola T, Nieminen T, Hartikainen S, Sulkava R, Vuolteenaho O, et al (2010) B-type natriuretic peptide as a predictor of declining cognitive function and dementia- a cohort study of an elderly general population with a 5-year follow-up. Ann Med 42: 207–215. 20384435 10.3109/07853891003652542

[19] Politi P, Minoretti P, Piaggi N, Brondino N, Emanuele E (2007) Elevated plasma N-terminal proBNP levels in unmedicated patients with major depressive disorder. Neurosci Lett 417: 322–325. 17350167 10.1016/j.neulet.2007.02.056

[20] Bunevicius R, Varoneckas G, Prange AJJ, Hinderliter AL, Fintauskiene V, et al (2006) Depression and thyroid axis function in coronary artery disease: impact of cardiac imapirment and gender. Clin Cardiol 29: 170–174. 16649727 10.1002/clc.4960290409 PMC6654096

[21] Jankowska EA, Drohomirecka A, Ponikowska B, Witkowska A, Lopuszanska M, et al (2010) Deficiencies in circulating testosterone and dehydroepiandrosterone sulphate and depression in men with systolic chronic heart failure. Eur J Heart Fail 12: 966–973. 20595194 10.1093/eurjhf/hfq108

[22] van den Broek KC, deFilippi CR, Christenson RH, Seliger SL, Gottdiener JS, et al (2011) Predictive value of depressive symptoms and b-type natriuretic peptide for new-onset heart failure and mortality. Am J Cardiol 107: 723. 21316507 10.1016/j.amjcard.2010.10.055 PMC3061261

[23] Almeida OP, Tamai S (2011) Congestive heart failure and cognitive functioning amongst older adults. Arq Neuropsiquiatr 59: 324–329. 10.1590/s0004-282x2001000300003 11460173

[24] Zuccala G, Cattel C, Manes-Gravina E, Di Niro MG, Cocchi A, et al (1997) Left ventricular dysfunction: a clue to cognitive impairment in older patients with heart failure. J Neurol Neurosurg Psychiatry 63: 509–512. 9343133 10.1136/jnnp.63.4.509 PMC2169754

[25] van Melle JP, de Jonge P, Ormel J, Crijns HJ, van Veldhuisen DJ, et al (2005) Relationship between left ventricular dysfunction and depression following myocardial infarction: data from the MIND-IT. Eur Heart J 26: 2650–2656. 16143708 10.1093/eurheartj/ehi480

[26] Bankier B, Barajas J, Martinez-Rumayor A, Januzzi JL (2008) Association between c-reactive protein and generalised anxiety disorder in stable coronary heart disease patients. Eur Heart J 29: 2212–2217. 18603621 10.1093/eurheartj/ehn326

[27] Bankier B, Barajas J, Martinez-Rumayor A, Januzzi JL (2009) Association between major depressive disorder and c-reactive protein levels in stable coronary heart disease patients. J Psychosom Res 66: 189–194. 19232230 10.1016/j.jpsychores.2008.09.010

[28] Bekelman DB, Havranek EP, Becker DM, Kutner JS, Peterson PN, et al (2007) Symptoms, depression and quality of life in patients with heart failure. J Card Fail 13: 643–648. 17923356 10.1016/j.cardfail.2007.05.005

[29] Mueller-Tasch T, Peters-Klimm F, Schellberg D, Holzapfel N, Juenger J, et al (2007) Depression is a major determinant of quality of life in patients with chronic systolic heart failure in general practice. J Card Fail 13: 818–824. 18068614 10.1016/j.cardfail.2007.07.008

[30] Vaes B, de Ruijter W, Degryse J, Westendorp RGJ, Gussekloo J (2009) Clinical relevance of a raised plasma N-terminal pro-brain natriuretic peptide level in a population-based cohort of Nonagenarians. J Am Geriatr Soc 57: 823–829. 19470010 10.1111/j.1532-5415.2009.02218.x

[31] Nilsson K, Gustafson L, Hultberg B (2010) The clinical use of N-terminal-pro brain natriuretic peptide in elderly patients with mental illness. Clin Biochem 43: 1282–1286. 20727348 10.1016/j.clinbiochem.2010.08.011

[32] Price JF, Reynolds RM, Mitchell RJ, Williamson RM, Fowkes FGR, et al (2008) The Edinburgh Type 2 Diabetes Study: study protocol. BMC Endocr Disord 8: 18–27. 19077235 10.1186/1472-6823-8-18 PMC2621220

[33] Hayashi C, Ogawa O, Kubo S, Mitsuhashi N, Onuma T, et al (2004) Ankle brachial pressure index and carotid intima-media thickness as atherosclerosis markers in Japanese diabetics. Diabetes Res Clin Pract 66: 269–275. 15536024 10.1016/j.diabres.2004.03.013

[34] Curb JD, Masaki K, Rodriguez BL, Abbott RD, Burchfiel CM, et al (1996) Peripheral artery disease and cardiovascular risk factors in the elderly- the Honolulu Heart Program. Arterioscler Thromb Vasc Biol 16: 1495–1500. 8977454 10.1161/01.atv.16.12.1495

[35] Marioni RE, Strachan MWJ, Reynolds RM, Lowe GDO, Mitchell RJ, et al (2010) Association between raised inflammatory markers and cognitive decline in elderly people with type 2 diabetes. Diabetes 59: 710–713. 19959761 10.2337/db09-1163 PMC2828661

[36] Deary IJ, Whalley LJ, Crawford JR (2004) An ‘instantaneous’ estimate of a lifetime's cognitive change. Intelligence 32: 113–119.

[37] Crawford JR, Dearyt IJ, Starr J, Whalley LJ (2001) The NART as an index of prior intellectual functioning: a retrospective validity study covering a 66-year interval. Psychol Med 31: 451–458. 11305853 10.1017/s0033291701003634

[38] Folstein MF, Folstein SE, McHugh PR (1975) ‘Mini-mental state’. A practical method for grading the cognitive state of patients for the clinician. J Psychiatr Res 12: 189–198. 1202204 10.1016/0022-3956(75)90026-6

[39] Zigmond AS, Snaith RP (1983) The hospital anxiety and depression scale. Acta Psychiatr Scand 67: 361–370. 6880820 10.1111/j.1600-0447.1983.tb09716.x

[40] Olsson I, Mykletun A, Dahl AA (2005) The hospital anxiety and depression rating scale: a cross-sectional study of psychometrics and case finding abilities in general practice. BMC Psychiatry 5: 46. 16351733 10.1186/1471-244X-5-46 PMC1343544

[41] Bjelland I, Dahl AA, Haug TT, Neckelmann D (2002) The validity of the hospital anxiety and depression scale: an updated literature review. J Psychosom Res 52: 69–77. 11832252 10.1016/s0022-3999(01)00296-3

[42] Spearman C (1904) ‘General intelligence’, objectively determined and measured. Am J Psychol 15: 201–292.

[43] Luciano M, Marioni RE, Gow AJ, Starr J, Deary IJ (2009) Reverse causation in the association between c-reactive protein and fibrinogen levels and cognitive ability in an aging sample. Psychosom Med 71: 404–409. 19398500 10.1097/PSY.0b013e3181a24fb9

[44] Deary IJ, Der G (2005) Reaction time explains IQ's association with death. Psychol Sci 16: 64–69. 15660853 10.1111/j.0956-7976.2005.00781.x

[45] Tombaugh TN, McIntyre NJ (1992) The mini-mental state examination: a comprehensive review. J Am Geriatr Soc 40: 922–935. 1512391 10.1111/j.1532-5415.1992.tb01992.x

[46] Reijmer YD, van den Berg I, Ruis C, Kappelle LJ, Biessels GJ (2010) Cognitive dysfunction in patients with type 2 diabetes. Diabetes Metab Res Rev 26: 507–519. 20799243 10.1002/dmrr.1112

[47] Carney RM, Freeland KE, Miller GE, Jaffe AS (2002) Depression as a risk factor for cardiac mortality and morbidity. J Psychosom Res 53: 897–902. 12377300 10.1016/s0022-3999(02)00311-2

[48] Thomas SA, Chapa DW, Friedmann E, Durden C, Ross A, et al (2008) Depression in patients with heart failure: prevalence, pathophysiological mechanisms and treatment. Crit Care Nurse 28: 40–55. 18378727

[49] Di Angelantonio E, Chowdhury R, Sarwar N, Ray KK, Gobin R, et al (2009) B-type natriuretic peptides and cardiovascular risk: systematic review and meta-analysis of 40 prospective studies. Circulation 120: 2177–2187. 19917883 10.1161/CIRCULATIONAHA.109.884866

[50] Wannamethee SG, Welsh P, Lowe GD, Gudnason V, Di Angelantonio E, et al (2011) N-terminal pro-brain natriuretic peptide is a more useful predictor of cardiovascular disease risk than c-reactive protein in older men with and without pre-existing cardiovascular disease. J Am Coll Cardiol 58: 56–64. 21700090 10.1016/j.jacc.2011.02.041

